# Renyi Distribution Entropy Analysis of Short-Term Heart Rate Variability Signals and Its Application in Coronary Artery Disease Detection

**DOI:** 10.3389/fphys.2019.00809

**Published:** 2019-06-26

**Authors:** Manhong Shi, Chaoying Zhan, Hongxin He, Yanwen Jin, Rongrong Wu, Yan Sun, Bairong Shen

**Affiliations:** ^1^Center for Systems Biology, Soochow University, Suzhou, China; ^2^College of Information and Network Engineering, Anhui Science and Technology University, Fengyang, China; ^3^Institutes for Systems Genetics, West China Hospital, Sichuan University, Chengdu, China

**Keywords:** coronary artery disease, heart rate variability, renyi distribution entropy, wavelet packet decomposition, classifier

## Abstract

Coronary artery disease (CAD) is a life-threatening condition that, unless treated at an early stage, can lead to congestive heart failure, ischemic heart disease, and myocardial infarction. Early detection of diagnostic features underlying electrocardiography signals is crucial for the identification and treatment of CAD. In the present work, we proposed novel entropy called Renyi Distribution Entropy (RdisEn) for the analysis of short-term heart rate variability (HRV) signals and the detection of CAD. Our simulation experiment with synthetic, physiological, and pathological signals demonstrated that RdisEn could distinguish effectively among different subject groups. Compared to the values of sample entropy or approximation entropy, the RdisEn value was less affected by the parameter choice, and it remained stable even in short-term HRV. We have developed a combined CAD detection scheme with RdisEn and wavelet packet decomposition (WPD): (1) Normal and CAD HRV beats obtained were divided into two equal parts. (2) Feature acquisition: RdisEn and WPD-based statistical features were calculated from one part of HRV beats, and student’s *t*-test was performed to select clinically significant features. (3) Classification: selected features were computed from the remaining part of HRV beats and fed into K-nearest neighbor and support vector machine, to separate CAD from normal subjects. The proposed scheme automatically detected CAD with 97.5% accuracy, 100% sensitivity and 95% specificity and performed better than most of the existing schemes.

## Introduction

Plaque accumulation (fatty and cholesterol substances) in the inner wall of the coronary arteries causes a blockage in the coronary circulation and the reduction of blood supply to the heart muscles, leading to coronary artery diseases (CAD) (Steinberg and Gotto, 1999). Unless treated early, CAD can result in congestive heart failure, ischemic heart disease, myocardial infarction, ischemia, arrhythmias, angina and sudden death (Grech, 2011). In 2012, 7.4 million CAD-related deaths were reported, which accounted for 10% of total fatalities among female population and 16% among male population that year, respectively (World Health Organization, 2015). By 2030, an estimated 37% increase in CAD-related death is expected in emerging nations (Acharya et al., 2017c). Early CAD detection is therefore the key to prevent further heart function damage and save lives.

The exercise stress test (EST), which monitors various heart status features, is often used for CAD diagnosis. However, not all CAD subjects can achieve the expected heart rate, and many patients may suffer cardiac arrest during EST (Román et al., 1998). Alternatively, measurement of resting ECG signals can be applied as a non-invasive and preferred method for CAD diagnosis. Since no obvious change in the resting ECG signals is detected among ∼70% of CAD subjects, the manual CAD diagnosis is time-consuming and ineffective (Antanavicius et al., 2008). In recent years, computer-aided diagnostic technologies (CADT) for CAD detection have garnered increasing attentions for their ease of operation without the excessive reliance on the personal experience of a doctor, as well as their cost-effectiveness.

Heart rate variability (HRV) extracted from the ECG depicts the variation in time interval between adjacent heartbeats and is vital for autonomic modulation of the heart. CADT-based HRV analyses have been proposed in recent years for CAD diagnosis. Reduced values in the frequency-domain feature of HRV signals is closely related to the severity of CAD (Hayano et al., 1990). For instance, compared to normal subjects, CAD patients exhibit lower circadian rhythms (Huikuri et al., 1994). Power spectral analysis also reveals that the low/high frequency ratio of HRV signals is significantly lower in CAD-affected subjects with panic disorder than in normal subjects (Lavoie et al., 2004). Moreover, CAD patients exhibit lower values in the time domain features of HRV signals, such as NN50 (number of adjacent NNs, which are greater than 50 ms) and pNN50 (NN50 divided by total number of NNs, which is expressed as a percentage), than normal subjects (Acharya et al., 2014). Due to its non-linear and non-stationary nature, the non-linear methods perform better at decoding the invisible complexities and extracting valuable information from HRV signals, compared to the frequency- and time-domain analyses of HRV signals. Applying non-linear methods can also minimize variation and background noise problems that are often associated with the frequency- and time- domain analyses. Many non-linear parameters, including the fractal dimension (Rajendra et al., 2005), the Lyapunov exponents (Acharya et al., 2004), the detrended fluctuation analysis (Peng et al., 1995), and the recurrence quantification analysis (RQA) (Acharya et al., 2014), are calculated from HRV signals in order to separate CAD from normal subjects.

Entropy, the main method of non-linear analysis that measures randomness and complexity of signals, is widely used for HRV signal analyses to detect cardiac abnormalities (Acharya et al., 2014, 2015b; Elias et al., 2014; Rajendra Acharya et al., 2015). Acharya et al. (2014) showed that Approximate Entropy (ApEn) and Sample Entropy (SamEn) are higher in normal subjects than in CAD subjects. Entropy evaluations are highly reliant upon the selection of parameters, including N (data length), r (distance tolerance), and m (embedding dimension) (Pincus, 1991; Mayer et al., 2014). Among these parameters, r has the largest impact on results, as even a small change in its value significantly alters the complexity measurement of a given data set, potentially causing mis-diagnosis (Castiglioni and Rienzo, 2008; Lu et al., 2008; Liu et al., 2011). Li et al. (2015) proposed a new entropy named the Distribution Entropy (DisEn) to measure the distribution property existing in data sets by computing Shannon entropy of the empirical probability density of inter-vector distances. Compared to the effect of r on ApEn and SamEn computations, the parameters M (the number of bins) and m (embedding dimension) have less impact on the stability and consistency of DisEn’s performance (Udhayakumar et al., 2016). DisEn excels in analyzing HRV signals with a shorter length (Karmakar et al., 2017). In contrast to the computation of ApEn and SamEn, which requires reconstruction of two adjacent dimension vector spaces, the computation of DisEn only requires reconstruction of the m-dimension vector space. As a result, the amount of DisEn computation is only half that of ApEn or SamEn. Renyi entropy (RenEn) is a generalization and reduction of Shannon entropy as the order parameter *q* closes to 1. RenEn, highlighting characteristics of multifractality or long-range interactions occurring in biomedical systems, is more sensitive to frequent occurrences when *q* increases (Liang et al., 2015). RenEn calculated from HRV signals is often used as an important clinical indicator of early cardiac autonomic neuropathy (Cornforth et al., 2013, 2014). Recently, RenEn has also been combined with non-linear decompositions, such as discrete wavelet transform (DWT) (Acharya et al., 2016), wavelet packet decomposition (WPD) (Li and Zhou, 2016), empirical mode decomposition (EMD) (Acharya et al., 2017b; Sridhar et al., 2017) for automated diagnosis of CAD, myocardial infarction, and congestive heart failure.

**FIGURE 1:**
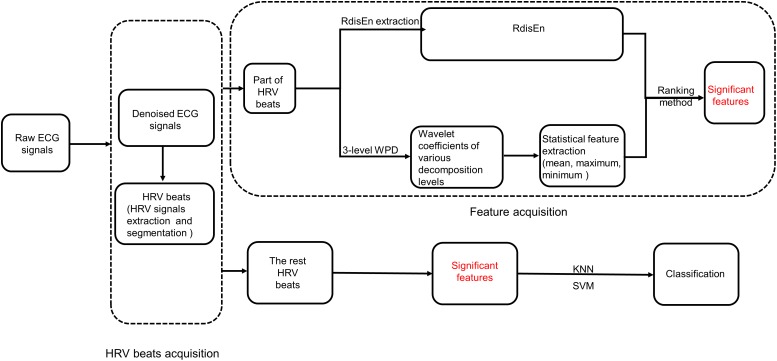
Block diagram of the scheme for CAD detection.

In our current studies, we propose a new entropy named Renyi Distribution Entropy (RdisEn), which integrates Renyi entropy and distribution entropy. In addition, we have developed an automatic CAD detection scheme (Figure 1): CAD and normal HRV beats are each divided into two parts. One part of HRV beats is used to extract features with important clinical information, while the other is used to evaluate classification performance of the CAD detection scheme. The feature extraction consists three steps: (1) CAD and normal HRV beats are subjected to three levels of WPD (2) Statistical features (mean, maximum, and minimum values) are computed from the obtained coefficients of the third decomposition levels, and RdisEn is computed from the HRV beats (3) The resulting WPD-based statistical features and RdisEn are then ranked to extract features with significant information for distinguishing those subjects with CAD from those without. Finally, the extracted features computed from the remaining part of HRV beats are fed into K-nearest neighbor (KNN) and support vector machine (SVM) for automatic CAD detection.

## Materials and Methods

### Data Acquisition

In this study, normal and CAD ECG recordings were downloaded from Fantasia open-access database^1^ and St. Petersburg Institute of Cardiological Technics 12-lead Arrhythmia Database^2^, respectively. Only the lead-II ECG recordings were used for this study. We employed a total of 57 ECG recordings, among which 40 were from normal subjects (20 old: 68 to 85 years, 20 young: 21 to 34 years) and 17 were from 7 CAD patients.

### Renyi Distribution Entropy

For a discrete time series {*x*(*i*), *i* = 1, 2,…, *N*}, *B* and *m* denote bin number and embedding dimension, the RdisEn is computed as follows:

(1) Reconstruction of state space: (*N*−*m*)vectors *U* (*i*) *byU* (*i*) = {*x* (*i*), *x*(*i*+1),…, *x*(*i*+*m*−1)}, 1 ≤ *i* ≤ N-m.

(2) Construction of the distance matrix: *M* = {*d_i,j_*} between vectors *U* (*i*) and *U* (*j*) for 1 ≤ *i, j* ≤ N-m, where

$$d_{i,j}=max\{|x(i+k)-x(j+k)|,\;0\leq k\leq m-1\}$$

(3) Estimation of probability density: the distances in the matrix M are divided into B bins with equal space, and thus the probability of each bin (t) of the histogram is calculated as

$$p_{t}=\frac{number\;of\;elements\;in\;the\;t\;bin}{total\;number\;of\;elements\;in\;M},\;t=1,\;2,\;\cdots ,\;B.$$

(4) Calculation: the normalized RdisEn of *x* (*i*) is defined as

$$RdisEn\;(B,\;m,\;q)=\frac{1}{(1-q)\;log_{2}^{\left(B\right)}}log_{2}\left(\sum_{t=1}^{B}p_{t}^{q}\right)$$

From the algorithm of RdisEn, it is not difficult to conclude that RdisEn will degenerate to DisEn when *q* → 1 (Cornforth et al., 2014).

### Proposed CAD Detection Scheme

In our present study, we developed a combination scheme to separate CAD patients and normal subjects. It consisted of three steps: (1) HRV beats acquisition, (2) RdisEn and WPD-based feature acquisition (3) classification through K-NN and SVM.

#### HRV Beats Acquisition

The downloaded ECG signals were sampled at 250 HZ for normal group and 257 HZ for CAD group. The normal ECG signals were up-sampled to 257 HZ to maintain uniformity between the two groups. Daubechies wavelet 6 (db6) was used to eliminate unwanted noise (Martis et al., 2013). The ECG signals were subjected to Pan-Tompkin to detect R-peaks (Pan and Tompkins, 2007), and then HRV signals were obtained by calculating the time duration of two consecutive R-peaks (Constant et al., 1999). Finally, each CAD HRV signal was divided into beats (each beat is a segment containing 500 samples), and as a result, 80 HRV beats were acquired from 17 normal ECG signals. In order to keep the dataset balance between the two groups, two normal HRV beats were extracted from each normal HRV signals, and consequently a total of 80 beats were acquired from 40 normal ECG signals.

#### Feature Acquisition

One hunderd and sixty HRV beats obtained from normal and CAD HRV signals were randomly divided into two parts before feature acquisition, with each part consisting of equal number of beats from the two classes. One part of beats (40 beats for each type of signals) was used for feature acquisition, while the other was utilized to evaluate classification performance.

##### Feature extraction

As shown in Figure 1, RdisEn and WPD-based statistical features were extracted from the HRV beats of the two classes. RdisEn was computed based on the distribution characteristics of inter-vector distances, and parameters on RdisEn evaluation were fixed at *B* = 512, *m* = 2, *q* = 0.4 for CAD detection in our proposed scheme (the rationale for the chosen combined parameter is detailed in section “Performance on CAD Detection”). RdisEn was used to extract significant features from each HRV beat to distinguish CAD patients from normal subjects, and WPD-based statistical features were also computed. Briefly, a 3-level WPD was performed on every segmented HRV beat with 500 samples to divide it into a set of sub-bands. WPD, a popular methodology of multiresolution analysis for non-stationary and non-linear signals (Acharya et al., 2015a), can exploit properties of the studied signals in frequency and time domains simultaneously. WPD provides better frequency resolution for the sub-bands than DWT, and it possesses more wavelet sub-bands on the 3rd level WPD of each HRV beat than DWT (8 for WPD and 4 for DWT). Since the obtained wavelet coefficients in each sub-band are related to the wavelet basis selected (Zhang et al., 2018), Daubechies (orders 1–5), Harr, Coiflets (orders 1–3) wavelet function with 3-level decomposition were considered to capture significantly discriminable features for the best classification accuracy for CAD detection. Subsequently, three statistical features, namely mean [*M(k)*], minimum [*Mi(k)*], and maximum [*Ma(k)*] (*k* = 1, 2…8,) were evaluated from the wavelet coefficients of the 3rd level wavelet sub-bands.

##### Feature selection

Not all of the features obtained from the HRV beats exhibited great separation between the two groups, redundant and insignificant features would raise computation cost and impede classification performance. To maximize classification accuracy, feature selection was applied to the original features from extraction, with the least number of features. Student’s *t*-test was used as a method of feature selection in our current study (Box, 1987), and features with a *p*-value less than 0.05 were deemed to have significant differences. RdisEn and the top four ranked statistical features were selected.

#### Classification

Top four ranked statistical features were computed from the remaining part of HRV beats using RdisEN, and fed into classifiers one by one to obtain the highest accuracy with minimum number of features. The two classifiers used in our work are described below.

##### K-nearest neighbor (KNN)

K-nearest neighbor is a supervised machine learning method widely used in classification, and the class of a testing data is determined by the majority of votes for *k* training samples with the closest Euclidean distance (González et al., 2016). In this work, *k* = 10 was used.

##### Support vector machine (SVM)

The SVM classifier divides the training set into two parts by constructing a hyper-plane in the feature space. Features in non-linear separation may be change into linear separation, using kernel functions to map the original data to a feature space with higher dimension (Duda et al., 2012). In this work, an order 1 function kernel was used.

Three evaluation indicators, Accuracy (Acc), Sensitivity (Sen), and Specificity (Spe) were calculated to evaluate the classifiers performance. To ensure unbiasedness and credibility of the classification results, 10 × 10-fold cross validation methodology was implemented, and overall evaluation indicators were calculated.

### Feature Assessment

Three statistics methods were used to test the CAD detection performance of these features. The open source R package was used for all the analysis and calculation. First, univariate binary logistic regression method was used to access the statistically significant correlations between CAD and each feature (*p*-value < 0.05). Second, the correlation between the extracted features was evaluated by using Pearson test (*p*-value < 0.05, correlation coefficient >0.5). Finally, multivariate binary logistic regression model without redundant features was established to determine statistically significant feature associated with CAD detection (*p*-value < 0.05).

## Results

### Performance of RdisEn on Various Signals

To test the consistency and stability of the RdisEn measurement, we studied the impact of the changing parameter combinations on the RdisEn measurement, using synthetic, physiologic and pathological signals. DisEn was originally introduced to eliminate ApEn and SamEn’s excessive dependence on tolerance *r*. RdisEn proposed in this work was based on DisEn. We therefore compared the performance of RdisEn to that of ApEn, SamEn and DisEn.

#### Performance of RdisEn on Synthetic Signals by Varying Parameters

**FIGURE 2:**
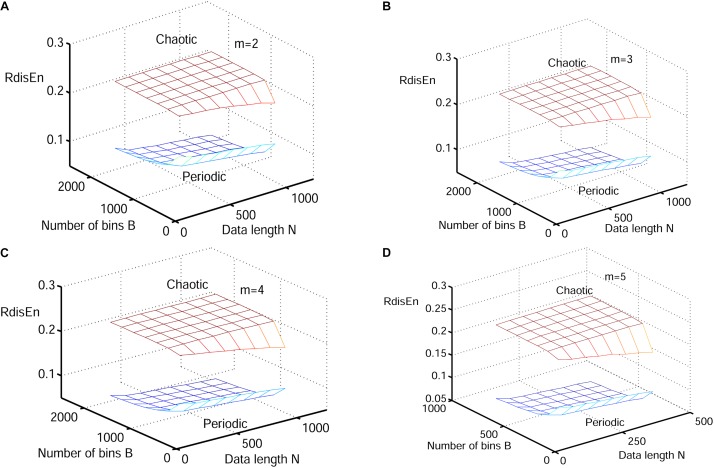
Variation of mean RdisEn values chaotic and periodic signals of with varying parameter combinations *N* and *B* for **(A)** *m* = 2, **(B)** *m* = 3, **(C)** m = 4, and **(D)** *m* = 5.

The synthetic signals were generated by the Logistic attractor *x*_*n*+1_ = *wx_n_* (1−*x_n_*). The constant w was set at 3.5 and 3.8 to obtain periodic and chaotic signals (Pincus, 1991) respectively, which has been widely applied to describe variations of entropy level (Xie et al., 2008; Chen et al., 2009; Karmakar et al., 2017). Twenty realizations were generated from 1000 samples of both signal types, and initial values of the realizations were selected randomly between 0.1 and 0.2 to eliminate the random factors. The mean values of RdisEn with the changing parameter combinations of *N (N* = 50, 200, 350, 500, 650, 800, 1000), *B (B* = 100,250,350,500,650,1000,1300,2000) and *m* (*m* = 2,3,4,5) for chaotic and periodic signals, as well as the fixed parameter *q* (*q* = 0.5), are shown in Figure 2. A significant separation between the two signal types was observed, while the traits of the RdisEn values were similar for *m* = 2, 3, 4, 5 (Figure 2).

#### Performance of RdisEn on Physiologic Signals by Varying Parameters

**FIGURE 3:**
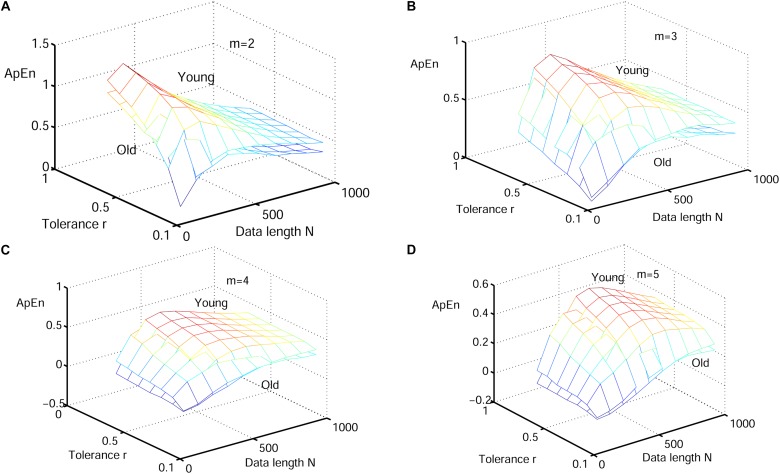
Variation of the mean ApEn value for HRV signals of old and young subjects with varying parameter combinations *N* and *r* for **(A)** *m* = 2, **(B)** *m* = 3, **(C)** *m* = 4, and **(D)** *m* = 5.

**FIGURE 4:**
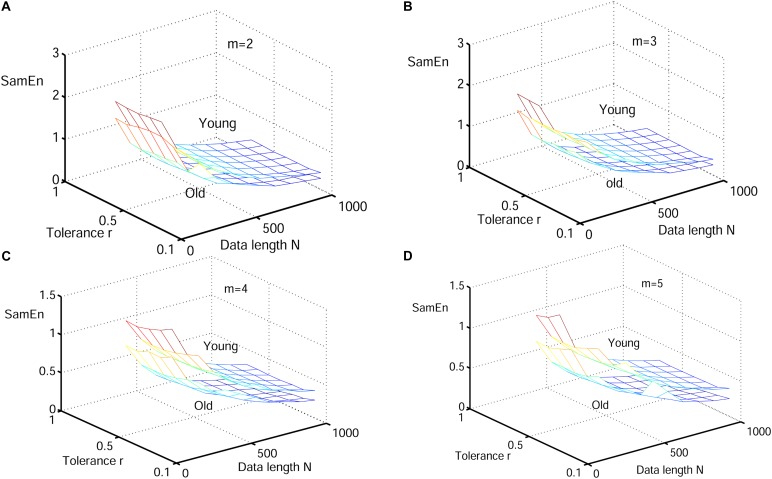
Variation of the mean value for HRV signals of old and young subjects with varying parameter combinations *N* and *r* for **(A)** *m* = 2, **(B)** *m* = 3, **(C)** *m* = 4, and **(D)** *m* = 5.

**FIGURE 5:**
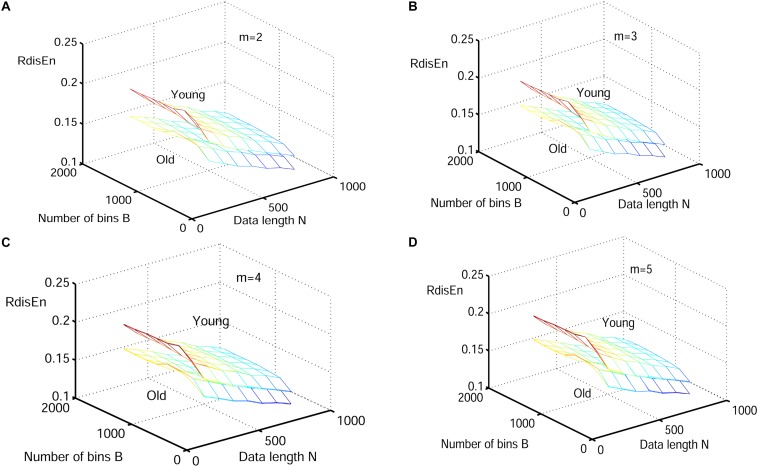
Variation of the mean RdisEn value for HRV signals of old and young subjects with varying parameter combinations *N* and *B* for **(A)** *m* = 2, **(B)** *m* = 3, **(C)** *m* = 4, and **(D)** *m* = 5.

Next, physiological features were extrapolated from the HRV signals of 20 elderly and 20 young healthy subjects as described in section “HRV Beats Acquisition.” For each subject, a HRV signal was selected with varying length (50, 200, 350, 500, 650, 800, and 1000) for RdisEn calculation using the following parameters: *N* = 50, 200, 350, 500, 650, 800, and 1,000; *B* = 100, 250, 350, 500, 650, 1,000, 1,300, and 2,000; *m* = 2, 3, 4, and 5; *q* = 0.5. As comparison, ApEn and SamEn were also calculated with the following parameters: *N* = 50, 200, 350, 500, 650, 800, and 1,000; *r* = 0.1^∗^*SD*, 0.2^∗^*SD*,…,1^∗^*SD*; *m* = 2, 3, 4, and 5. The results are shown in Figure 3–5. The values of ApEn fluctuated widely with different combinations of *N, r*, and *m*, especially for a small data length (Figure 3). There was a crossover in ApEn meshes between the HRV signals from the elderly and young subjects, suggesting that ApEn failed to effectively separate the two age groups. The SamEn mesh was sparse in comparison with the ApEn and RdisEn mesh (Figure 4), most likely due to the fact that SamEn was not defined for smaller data length, resulting in invalid values. As shown in Figure 5, RdisEn could effectively differentiate HRV signals between the two age groups, even for smaller data lengths. In addition, the variation of RdisEn values was small with diverse parameter *m*. The effects of different parameters *N* and *B* or *r* on entropy measurements (ApEn and RdisEn) for embedding dimension *m* ∈ [2, 5] were quantified by the means of the standard deviation across *N* and *B* or r (Table 1). It was not difficult to find that the change of ApEn measurement with a variation of N was less than that with the change of *r*. The variation of RdiEn measurement with varying B was higher than that with varying of N for old vs. young subjects. More importantly, compared to ApEn, the variations of RdisEn values with changing parameters *B* and *M* were relatively small (Table 1).

**Table 1:** Mean of the standard deviation across data length *N* and bin number *B* for RdisEn or tolerance *r* for ApEn.

Entropy	Embedding dimension m	Young		Old		Healthy		CAD		Healthy		Arrhythmia	
							
		$\bar{\sigma_{N}}$	$\bar{\sigma_{B/r}}$	$\bar{\sigma_{N}}$	$\bar{\sigma_{r/B}}$	$\bar{\sigma_{N}}$	$\bar{\sigma_{r/B}}$	$\bar{\sigma_{N}}$	$\bar{\sigma_{r/B}}$	$\bar{\sigma_{N}}$	$\bar{\sigma_{r/B}}$	$\bar{\sigma_{N}}$	$\bar{\sigma_{r/B}}$
RdisEn	2	0.0043	0.0242	0.0043	0.0217	0.0043	0.0217	0.0067	0.0226	0.004	0.0192	0.0074	0.0196
	3	0.0048	0.0244	0.0049	0.0219	0.0049	0.0219	0.0079	0.0229	0.0051	0.0195	0.0084	0.0199
	4	0.0054	0.0245	0.0054	0.0220	0.0054	0.0220	0.0087	0.0231	0.0057	0.0196	0.0093	0.0200
	5	0.0060	0.0245	0.0060	0.0221	0.0060	0.0221	0.0096	0.0221	0.0062	0.0197	0.0101	0.0201
ApEn	2	0.1302	0.2641	0.0982	0.2527	0.0982	0.2527	0.0404	0.1225	0.0829	0.2481	0.1063	0.2322
	3	0.1114	0.1893	0.0859	0.1599	0.0859	0.1599	0.0401	0.0698	0.0741	0.1487	0.0995	0.1357
	4	0.0983	0.1687	0.0770	0.1314	0.0770	0.1314	0.0447	0.0419	0.0657	0.1122	0.0867	0.11104
	5	0.0875	0.1545	0.0707	0.1172	0.0707	0.1172	0.0447	0.0316	0.0578	0.0941	0.0748	0.0995


#### Performance of RdisEn on Pathological Signals by Varying Parameters

In this work, CAD signals and arrhythmia short-term HRV signals were compared to healthy signals. 40 healthy and 17 CAD short-term HRV signals were acquired from 40 normal and 7 CAD subjects, as described in section “HRV Beats Acquisition.” Using the Pan-Tompkin algorithm, 48 arrhythmia short-term HRV signals were obtained from MIT-BIH Arrhythmia Database^3^, which contains 48 ECG signals from 47 subjects (25 men: 32 to 89 years, 22 women: 23 to 89 years) (Pan and Tompkins, 2007).

The HRV signals of varying lengths (50, 200, 350, 500, 650, 800, and 1,000) were selected to test ability of RdisEn to separate the two types of pathological signals with changing parameters. ApEn and SampEn were also calculated as references, and the parameters corresponding to the three entropies (RdisEn, ApEn, and SamEn) were set as described in section “Performance of RdisEn on Physiologic Signals by Varying Parameters.”

**FIGURE 6:**
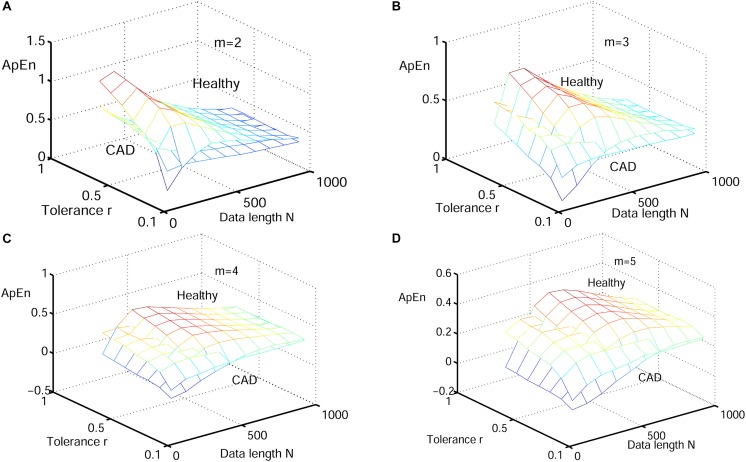
Variation of the mean ApEn value for HRV signals of normal and CAD subjects with varying parameter combinations *N* and *r* for **(A)** *m* = 2, **(B)** *m* = 3, **(C)** *m* = 4, and **(D)** *m* = 5.

**FIGURE 7:**
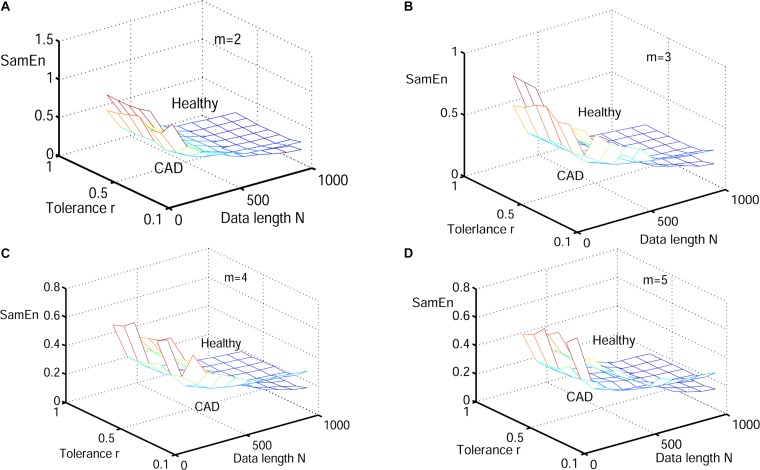
Variation of the mean SamEn value for HRV signals of normal and CAD subjects with varying parameter combinations *N* and *r* for **(A)** *m* = 2, **(B)** *m* = 3, **(C)** *m* = 4, and **(D)** m = 5.

**FIGURE 8:**
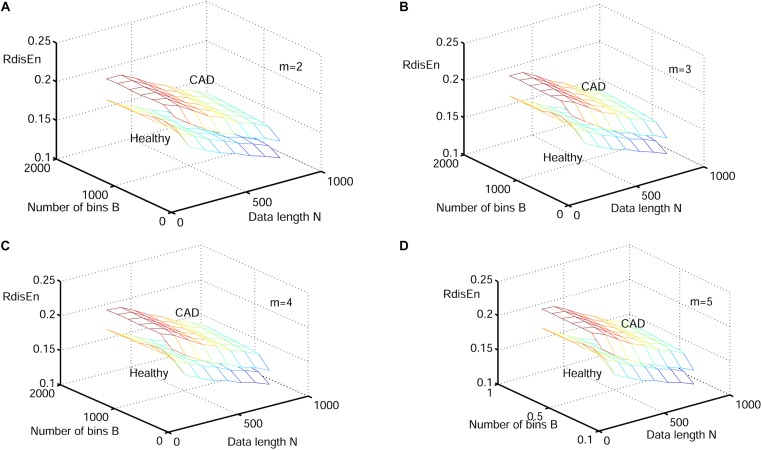
Variation of the mean RdisEn value for HRV signals of normal and CAD subjects with varying parameter combinations *N* and *B* for **(A)** *m* = 2, **(B)** *m* = 3, **(C)** *m* = 4, and **(D)** *m* = 5.

Figure 6–8 show the change of mean ApEn, SamEn and RdisEn values with varying parameter combinations for normal and CAD HRV signals. In contrast to ApEn and SamEn, the mean value of RdisEn for CAD HRV signals was higher than that for normal HRV signals, demonstrating the superiority of RdisEn in differentiating CAD and normal subjects. Quantification results on the effect of parameter selections for entropy values (ApEn and RdisEn) for embedding dimension *m* ∈ [2, 5], in terms of the means of standard deviation across *N* and *B* or r, are shown in Table 1. Our results demonstrated that RdisEn measurement remained relatively stable to parameter selection for very short lengths of HRV signals (Figure 3–8 and Table 1), most likely due to its inherited merits from DisEn (Karmakar et al., 2017). In contrast, a large amount of invalid values was generated in ApEn and SamEn calculation (Figure 6, 7). Moreover, remarkable separation between normal and CAD RdisEn meshes was observed (Figure 8). Taken together, these results demonstrated that the RdisEn analysis is the best method to generate stable values with clear separation between normal and CAD groups.

**FIGURE 9:**
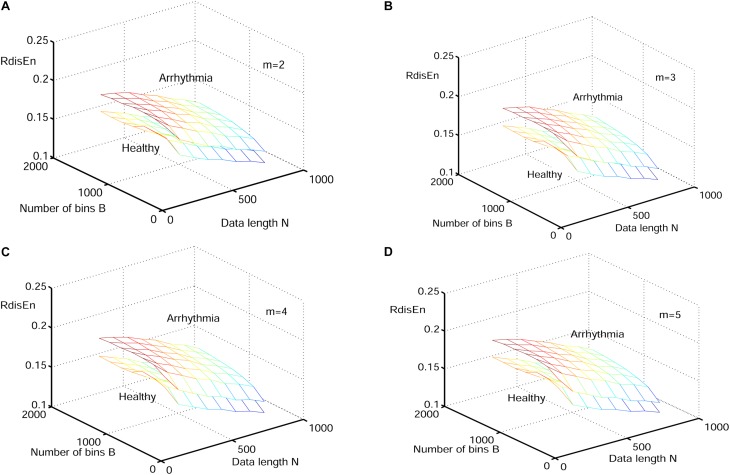
Variation of the mean RdisEn value for HRV signals of normal and arrhythmia subjects with varying parameter combinations *N* and *B* for **(A)** *m* = 2, **(B)** *m* = 3, **(C)** *m* = 4, and **(D)** *m* = 5.

The change of mean RdisEn values with varying parameter combinations for normal and arrhythmia HRV signals was shown in Figure 9. The parameter combinations of RdisEn were the same as that described in section “Performance of RdisEn on Physiologic Signals by Varying Parameters.” Significant separation between normal and arrhythmia RdisEn meshes can be observed (Figure 9). In addition, area under the ROC curve (AUC) was used to examine the performance of RdisEn (*B* = 512, *m* = 2, *q* = 0.9) with varying length (500, 800, and 1000) to separate healthy from arrhythmia short-term HRV signals. ApEn (*r* = 0.2 × *SD, m* = 2), SamEn (*r* = 0.2 × *SD, m* = 2), DisEn (*B* = 512, *m* = 2), and RenEn (*q* = 0.9) were used as references. When AUC equals to 1, the feature distributions belonging to the two classes are completely separated; when AUC equals to 0.5, the feature distributions are similar, suggesting that the closer to 1 the AUC value, the better the discriminatory power of RdisEn (Hanley and McNeil, 1982). RisEn out-performed ApEn, SamEn, DisEn, and RenEn in distinguishing healthy from arrhythmia short-term HRV signals (Table 2 and Figure 9). RdiEn also exhibited good computing stability with varying length, as shown by the SD values (Table 2).

**Table 2:** AUC values of the five entropy measurements with varying lengths for separating healthy from arrhythmia HRV signals.

Entropy	500	800	1000	Mean	SD
ApEn	0.4427	0.4552	0.4583	0.4521	0.0083
SampEn	0.5833	0.5729	0.551	0.5691	0.0165
DisEn	0.7719	0.7677	0.7604	0.7667	0.0058
RenEn	0.5208	0.5104	0.45	0.4937	0.0382
RdisEn	0.7698	0.7698	0.7656	0.7684	0.0024


### Performance on CAD Detection

We repeated the RdisEn analyses using HRV beats obtained via the automated CAD detection scheme from normal and CAD subjects. *B* = 512 was frequently employed in the analysis of HRV signals with respect to DisEn (Li et al., 2015; Yang et al., 2015), and the selection of *m* had little effect upon the RdisEn evaluation on the basis of the aforementioned study. Consequently, the related parameters on the RdisEn evaluation were fixed to *B* = 512 and m = 2 for CAD detection. In addition to the parameter *B* and *m* on the DisEn evaluation, another parameter *q*, which enhances differentiation between normal and CAD HRV beats, requires to be fixed for the RdisEn measurement. To optimize *q*, we adopted the Student’s *t-*test to assess the performance of RdisEn calculated from one part of normal and CAD HRV beats with varying parameter *q* (*q* ∈ [0.1, 2]). As shown in Table 3, the two groups exhibited significantly different RdisEn values, regardless of the *q* parameter variations. The *p*-value was lowest when *q* = 0.4, indicative of the optimal condition to differentiate the two groups (RdisEn degenerated into DisEn, as mentioned in section “Renyi Distribution Entropy”). As a result, we computed RdisEn with parameters *N* = 500, *B* = 512, *m* = 2, and *q* = 0.4. Table 4 shows the means and SD of RdisEn, as well as the top four statistical features based on WPD with the db1, computed from one part of normal and CAD HRV beats, and their *p*-values generated by Student’s *t-*test, significant differences were observed between the two groups (Table 4).

**Table 3:** *p*-Values of RdisEn computed from normal and CAD HRV beats with varying parameter *q.*

*q*	0.1	0.2	0.3	0.4	0.5	0.6	0.7	0.8	0.9	1
*p*-Value	9.68E-5	5.08E-5	3.55E-5	3.15E-5	3.32E-5	3.85E-5	4.68E-5	5.60E-5	7.1E-5	8.86E-5

*q*	1.1	1.2	1.3	1.4	1.5	1.6	1.7	1.8	1.9	2
*p*-Value	1.08E-4	1.30E-4	1.54E-4	1.81E-4	2.09E-4	2.39E-4	2.71E-4	3.05E-4	3.39E-4	3.74E-4


**Table 4:** Mean and SD values of RdisEn and the top five WPD (db1 basis) based statistical features for normal and CAD HRV beats.

Feature	CAD		Normal		*p*-value
			
	Mean	SD	Mean	SD	
RdisEn	0.2690	0.0363	0.2345	0.0316	3.15E-5
Mi(1)	1.6203	0.3024	2.4613	0.4826	2.37E-14
M(1)	1.9249	0.3958	2.8147	0.5110	3.55E-13
Mi(5)	2.2415	0.4469	3.0369	0.5778	1.29E-9
Ma(6)	0.3315	0.2148	0.0992	0.0995	2.45E-8


Five selected features were calculated from the remaining part of normal and CAD HRV beats and then fed into classifiers KNN or SVM one by one to maximize the accuracy with minimal features. As shown in Table 5, the proposed scheme for CAD detection achieved the highest mean accuracy of 96.34% with five features (RdisEn and four WPD with db1 d statistical features) in 10 × 10-fold cross validation using KNN. We repeated the CAD detection scheme (Figure 1) using RdisEn and WPD with other wavelet-based statistical features, and the results were shown in Table 5. We computed the *p*-values for the correlations between CAD detection with five features, i.e., RdisEn and the top ranked four WPD with coif2 based statistical features [Mi(1), M(1), Ma(1), and Mi(6)] by univariate binary logistic regression method, the corresponding *p*-values for the five features are 2.5e-6, 0.01, 3.3e-6, 4.6e-6, and 0.02, respectively, which demonstrated that these features statistically significant correlated with CAD detection. As presented in Table 5, when the fifth feature was added into the KNN classifier, the CAD detection performed with 97.5% accuracy, 100% sensitivity, 95% specificity, respectively, with no significant improvement compared with the performance of the 4-feature based model (97.37 ± 0.41% accuracy, 99.75 ± 0.79% sensitivity, 95% specificity). This indicated that the fifth feature [Mi(6)] is correlated with the other four features [RdisEn, Mi(1), M(1), and Ma(1)] and therefore includes redundant information. A Pearson test was performed to calculate the correlations between the fifth feature and other four, the correlation coefficient for Mi(6) and RdisEn, fMi(1), M(1), and Ma(1) are -0.26 (*p*-value = 0.020), -0.073 (*p*-value = 0.518), 0.218 (*p*-value = 0.052), and 0.953 (*p*-value = 4.3e-42), respectively, indicating a significant correlation between the fifth feature [Mi(6)] and Ma(1). At last, the multivariate binary logistic regression model was used to test the relationship between the features and the CAD detection, in which RdisEn and the other three features were entered and their *p*-values are 0.033, 0.563, 0.089, and 0.501, respectively; it is obvious that RdisEn improved the CAD detection statistically significant.

**Table 5:** Classification performance of RdisEn and WPD (various basis) based statistical features by using KNN and SVM classifiers.

Wavelet basis	NoF	KNN			SVM		
			
		Acc(%)	Sen(%)	Spe(%)	Acc(%)	Sen(%)	Spec(%)
db1	3	96.08 ± 0.38	97.5	94.75 ± 0.79	96.08 ± 0.38	97.25 ± 0.79	95
	4	96.08 ± 0.38	97.5	94.75 ± 0.79	96.06 ± 0.59	97.5 ± 1.79	95.24 ± 0.77
	5	96.34 ± 0.72	97.5	95.25 ± 1.42	95.6 ± 0.85	97.25 ± 0.79	94 ± 1.29
db2	3	96.08 ± 0.38	97.5	94.75 ± 0.79	97.12 ± 0.84	99.25 ± 1.69	95
	4	97.25 ± 0.79	100	94.5 ± 1.58	97.37 ± 0.41	99.75 ± 0.79	95
	5	96.48 ± 0.99	98 ± 1.97	95	96.6 ± 0.86	98.25 ± 1.69	95
db3	3	96.33 ± 0.41	97.75 ± 0.79	95	95.6 ± 0.85	96.5 ± 1.75	94.75 ± 0.79
	4	97.37 ± 0.41	99.75 ± 0.79	95	96.6 ± 0.86	98.5 ± 1.75	94.75 ± 0.79
	5	95.72 ± 0.62	96.5 ± 1.29	95	96.46 ± 0.54	98 ± 1.05	95
db4	3	95.96 ± 0.51	97 ± 1.05	95	95.38 ± 1.31	95.75 ± 2.65	95
	4	97.37 ± 0.41	99.75 ± 0.79	95	96.23 ± 1.17	97.5 ± 2.36	95
	5	97.37 ± 0.41	99.75 ± 0.79	95	95.24 ± 0.95	95.5 ± 1.97	95
db5	3	95.84 ± 0.58	97 ± 1.05	94.75 ± 0.79	95 ± 1.26	95 ± 2.63	95
	4	96.23 ± 1.02	97.75 ± 0.79	94.75 ± 1.84	95.85 ± 0.83	97 ± 1.05	94.75 ± 1.42
	5	96.74 ± 1.22	98.75 ± 1.32	94.75 ± 1.84	95.60 ± 1.34	96.75 ± 1.69	94.50 ± 1.58
db6	3	95.96 ± 0.51	97.50	94.50 ± 1.05	95.97 ± 0.78	97 ± 1.58	95
	4	97.24 ± 0.55	100	94.5 ± 1.05	96.73 ± 0.89	98.50 ± 1.75	95
	5	95.85 ± 0.83	97.50 ± 1.18	94.25 ± 1.21	95.48 ± 0.84	96.50 ± 1.75	94.50 ± 1.05
harr	3	96.08 ± 0.38	97.50	94.75 ± 0.79	96.08 ± 0.38	97.25 ± 0.79	95
	4	96.08 ± 0.38	97.50	94.75 ± 0.79	96.21 ± 0.59	97.50 ± 1.18	95.24 ± 0.77
	5	96.34 ± 0.71	97.50	95.25 ± 1.42	95.60 ± 0.85	97.25 ± 0.79	94 ± 1.23
coif1	3	96.08 ± 0.38	97.50	94.75 ± 0.79	95.84 ± 0.81	97 ± 1.05	94.75 ± 0.79
	4	95.96 ± 0.51	97.50	94.50 ± 1.05	95.85 ± 1.01	97.25 ± 1.42	94.50 ± 1.05
	5	97.24 ± 0.55	99.75 ± 0.79	94.75 ± 0.79	96.24 ± 1.31	98 ± 1.97	94.50 ± 1.05
coif2	3	96.33 ± 0.41	97.75 ± 0.79	95	97.11 ± 0.63	99.25 ± 1.21	95
	4	97.37 ± 0.41	99.75 ± 0.79	95	97.24 ± 0.55	99.5 ± 1.05	95
	5	97.50	100	95	97.24 ± 0.55	100	94.50 ± 1.05
coif3	3	95.96 ± 0.51	97.25 ± 0.79	94.75 ± 0.79	96.98 ± 0.98	97 ± 1.97	95
	4	96.98 ± 0.67	99.25 ± 1.21	94.75 ± 0.79	96.99 ± 0.88	99 ± 1.75	95
	5	95.84 ± 0.58	96.75 ± 1.21	95	95.36 ± 0.58	95.75 ± 1.21	95


*NoF, number of features.*

## Discussion

The CAD diagnostic signals can be divided into three major categories: heart sound signals (HSS), ECG signals, and HRV signals. The detailed methods of CAD detection are described in Table 6. HRV signals are essential tools widely used for cardiac abnormality detection and CAD diagnosis (Lee et al., 2007, 2008; Dua et al., 2012; Giri et al., 2013; Patidar et al., 2015; Kumar et al., 2016). Lee et al. (2007, 2008) employed linear and non-linear features extracted from HRV signals as indices to distinguish normal subjects from CAD patients, and they achieved a ∼90% accuracy using the SVM classifier. The automatic CAD detection algorithm was proposed by Dua et al. (2012), based on non-linear features (recurrence plots, detrended fluctuation, and three types of entropy) and a principal component analysis method, and achieved an accuracy of 89.5% using multilayer perceptron (MLP) methodology. Giri et al. (2013) used DWT to divide HRV signals into frequency sub-bands. They also applied dimensionality reduction methods such as PCA, independent component analysis (ICA), and linear discriminant analysis (LDA) to the coefficients from the obtained sub-bands to lower the data dimension. With the additional combined method of ICA and Gaussian mixture model (GMM), Giri et al. (2013) reported the highest accuracy for automatically identifying CAD of 96.8%. Patidar et al. (2015) applied a combination of tunable-Q wavelet transform (TQWT), centered correntropy, and the PCA method to achieved an accuracy of 99.72% with HRV signals from the automated diagnosis of CAD. Kumar et al. (2016) developed a CAD detection technique, which consists of the flexible analytic wavelet transform (FAWT) and ranking methods, including receiver operating characteristics (ROC), entropy and Bhattacharya space algorithm, with a classification accuracy of 100%.

**Table 6:** Studies conducted to distinguish normal from CAD subjects using various signals.

Author	Data used	Method/features	Classifiers	Cross validation	Accuracy
**using HSS signals**					
Karimi et al., 2005	5 CAD and 5 normal	DWT, WPD (some statistical features)	ANN	No	90%
Zhao and Ma, 2008	40 CAD and 40 normal	EMD, TEO (some statistical features)	BPNN	No	85%
**using ECG signals**					
Lewenstein, 2001	479 CAD and 297 normal	(slope of an ST segment, blood pressure, load during the test)	Radial basis function neural networks	No	97%
Babaoǧlu et al., 2010a	480 CAD	principle component analysis	SVM	5-fold	79.1%
Babaoglu et al., 2010b	480 CAD	binary particle swarm optimization and genetic algorithm	SVM	5-fold	81.46%
Acharya et al., 2017c	7 CAD and 40 normal	Higher-Order Statistics and Spectra (HOS)	KNN,DT	10-fold	98.99%
Kumar et al., 2017	7 CAD and 40 normal	Flexible analytic wavelet transform (cross information potential)	LS-SVM	10-fold	99.6%
**Normal and CAD using HRV signals**					
Lee et al., 2007	99 CAD, 94 Normal	Linear (time domain, frequency domain) and non-linear methods (Poincare plot, approximation entropy)	Support vector machine (SVM)	10-fold	90%
Lee et al., 2008	99 CAD, 94 Normal	Linear (time domain, frequency domain) and non-linear methods (Poincare plot, the hurst exponent, Detrended fluctuation analysis)	CPAR & SVM:	10-fold	85–90%
Dua et al., 2012	10 CAD and 10 normal subjects	Non-linear methods (recurrence plots, Shannon entropy) and principal component analysis (PCA)	multilayer perceptron (MLP)	5-fold	89.5%
Giri et al., 2013	10 CAD and 15 normal subjects	DWT and Independent Component Analysis (ICA)	Gaussian Mixture Model (GMM)	3-fold	96.8%
Patidar et al., 2015	10 CAD and 10 normal subjects	TQWT and PCA (correntropy)	LS-SVM	3-fold	99.72%
Kumar et al., 2016	10 CAD and 10 normal subjects	FAWT and entropy	LS-SVM	10-fold	100%
**In this work**	40 normal and 7 CAD subjects	RdisEn and WPD (statistical features)	KNN and SVM	10 times 10-fold	97.5%


In the current study, we proposed a new entropy called RdisEn based on DisEn. Our simulation experiments with three types of signals showed that RdisEn, as an indicator of the randomness and complexities occurring in the signals, was not overly reliant on parameter selection and remained stable for even very short sequences (Figure 5, 8). It out-performed ApEn and SamEn in separating physiological (old from young) and pathological (healthy from CAD and healthy from arrhythmia) signals (Figure 3–8). The results indicate that RdisEn is a promising measure to characterize physiological and pathological condition of subjects with short-term HRV signals.

We developed an automatic CAD detection scheme combining RdisEn and WPD-based statistical features to analyze short-term HRV signals. Since the HRV signals used in this work were extracted from standard ECG signals obtained during the rest period instead of exercise, the ECG signal acquisition were harmless to the test subjects. Using only five features with KNN and the 10 × 10-fold cross validation method, the proposed scheme can differentiate normal and CAD affected HRV with 97.5% accuracy, demonstrating that our scheme outperformed other algorithms in automatic CAD detection (Table 5). It was worth mentioning that, in feature acquisition, features were extracted from a data set, which was independent of that used for the subsequent evaluation of the classifier in our work. Compared with other CAD detection schemes shown in Table 6, our scheme using RdisEn and WPD-based statistical features is more stable, rigorous and efficient. The classification accuracy achieved was significantly higher than that using WPD-based statistical features alone (97.5% vs. 90%) (Karimi et al., 2005). The *p*-values of 2.5e-6 and 0.033 for RdisEn by univariate and multivariable binary logistic regression method, respectively, were obtained in the process of testing the ability of RdisEn as a feature for CAD detection in this work. These indicated that RdisEn made a great contribution in distinguishing normal and CAD affected HRV signals. In the future, RdisEn can be utilized as a quantification index of irregularity within non-linear signals for the diagnosis of other diseases such as fibrillation, myocardial infarction and congestive heart failure (Acharya et al., 2017a, 2018a,b; Fujita and Cimr, 2019).

## Conclusion and Future Work

Coronary artery disease is a serious cardiac abnormality, leading to high fatality. Early diagnosis and treatment of CAD can prevent progression. In this work, we proposed a new important entropy named RdisEn. It can effectively reveal the irregularity and randomness in HRV beats. A scheme for the automated differentiation between HRV signals from normal and CAD affected people has been developed, using WPD- and RdisEn-based computation, Student’s *t*-test selection, and classifiers to yield a classification accuracy of 97.5%, sensitivity of 100% and specificity of 95%. This novel scheme for CAD detection is reproducible, cost-effective, non-invasive, and more accessible than physical examinations such as coronary angiography and cardiac catheterization. In future works, we will apply the proposed scheme for the diagnosis of CAD and test this model in big population samples for future application.

## Data Availability

Publicly available datasets were analyzed in this study. This data can be found here: https://www.physionet.org/physiobank/database/incartdb/.

## Author Contributions

MS and CZ contributed to the majority of writing and conducted major parts of the experiments. YJ, HH, RW, and YS conducted some experiments and contributed to the methodology and writing. BS supervised the project and revised the manuscript.

## Conflict of Interest Statement

The authors declare that the research was conducted in the absence of any commercial or financial relationships that could be construed as a potential conflict of interest.

## Footnotes

1 https://physionet.org/physiobank/database/fantasia/

2 https://www.physionet.org/physiobank/database/incartdb/

3 https://physionet.org/physiobank/database/mitdb/
